# Intravenous Iron Technique Evaluation in Chronic Heart Failure With Iron Deficiency Anemia

**DOI:** 10.7759/cureus.47778

**Published:** 2023-10-27

**Authors:** Rohit Gosavi, Nitin B Jadhav, Abhijeet Nashte

**Affiliations:** 1 Department of Medicine, Krishna Vishwa Vidyapeeth, Karad, IND

**Keywords:** coronary heart disease, hemoglobin levels, true anemia, inflammatory cytokines, heart failure, erythropoiesis-stimulating agents, iron deficiency, chronic heart failure

## Abstract

Objective:This study aimed to investigate and assess whether IV iron improves symptoms of chronic heart failure (CHF) in patients with iron deficiency anemia (IDA).

Method:A total of 66 subjects with heart failure (HF) seeking therapy in the Department of Medicine's Inpatient Department (IPD) and Outpatient Department (OPD) were included. The data were collected during an outpatient or inpatient visit, documented in a predesigned and pretested proforma and then evaluated. All subjects received history-taking, examinations and regular laboratory tests after being informed and signing an agreement. On admission, the following data was collected: name, age, gender and comorbidities. The examination of subjects included a general examination and a systematic examination. Hematological parameters including hemoglobin (Haemometer, Top Tech Bio Medicals Mumbai), serum iron (Roche Cobas c501, USA), total iron binding capacity (TIBC, Beckman Coulter AU480, India), transferrin saturation percentage (TSAT% = (serum iron/TIBC) × 100), left ventricular ejection fraction (LVEF, 2D echocardiography, Nivan Healthcare Solutions, India) and ferritin (Abbott Architect Ferritin Assay, Delhi) are also important. Other blood tests like liver and renal function tests include an electrocardiogram (12-lead ECG) and two-dimensional echocardiography on admission and follow-up.

Results: In our study, 66 patients in total received IV iron as a treatment option to improve the symptoms of CHF with IDA; the New York Heart Association (NYHA) classification showed significant improvement (p-value <0.001). Before the intervention, 57.58% of patients had NYHA class II and 42.4% of patients had NYHA class III. After treatment, 33.33% of patients showed NYHA class II and 19.70% of patients showed NYHA class III. After iron therapy treatment, out of 29 cases of NYHA class III, nine (31.03%) cases converted to NYHA class I, seven (24.14%) cases converted to NYHA class II, and 13 (44.83%) cases belonged to the same NYHA class. Out of 37 cases of NYHA class II, 22 (59.45%) cases converted to NYHA class I, and 15 (40.54%) cases belong to the same NYHA class.

Conclusion:Thus, we come to the conclusion that the NYHA classification has exhibited notable enhancement subsequent to the administration of parenteral iron therapy. Sufficient evidence exists to substantiate the advantageous effects of intravenous iron therapy in the treatment of iron deficiency anemia. The administration of iron therapy has been observed to yield favorable outcomes in the mitigation of symptoms among individuals afflicted with cardiac insufficiency.

## Introduction

Many studies have shown that despite receiving excellent conventional treatment, many patients with chronic heart failure (CHF) remain asymptomatic, exercise intolerant and have high rates of hospitalization and mortality [1,2]. It has been shown by studies that iron deficiency anemia (IDA) is most commonly seen in patients with CHF along with unfavorable effects [1,2]. In the past, the presence of iron deficiency anemia was the sole clinical indicator of iron deficiency (ID). Almost one-third of patients with CHF without anemia can have IDA, despite the fact that iron deficiency anemia is often found in anemic CHF patients [1,2]. Up to half of all episodes of anemia are thought to be caused by IDA, making it the most common kind of anemia worldwide [1,2]. Studies in the past have also concluded that IDA with heart failure (HF) can lead to poor cardiac function, myocardial contractility and renal function which can lead to morbidity and mortality in the patients. It is found that patients who have had cardiac disease in the past (with increasing age and chronic cardiac disease) can present with signs and symptoms of cardiac failure in the absence of clinically apparent iron deficiency anemia. Hence, studies have also shown that, in early iron deficiency conditions, anemia is not detected, so for that reason, the initiation of intravenous iron therapy should be started, which can result in symptomatic improvement and even improve cardiac function in heart failure patients [2].

More recently, studies found that administration of intravenous iron in patients with heart failure and absolute or functional iron deficiency with or without anemia improves symptoms and exercise capacity [3]. Furthermore, studies also concluded that if anemia and IDA are indeed mediators of poor outcomes in patients with HF, then correcting these comorbidities would be necessary, and thus, novel therapeutic agents are required to improve outcomes. Several small studies show that the use of erythropoiesis-stimulating agents (ESAs) to increase hemoglobin in patients with HF with reduced ejection fraction is associated with beneficial effects on clinical outcomes [4,5]. Additionally, researchers have also concluded that hemoglobin levels are reduced to below-optimal levels when iron deficiency occurs [6,7].

Studies also revealed that the prevalence of anemia in patients with HF (defined as hemoglobin <13 g/dl in men and <12 g/dl in women) [8] is ≈30% in stable patients and ≈50% in hospitalized patients. Regardless of whether patients have heart failure with preserved ejection fraction (HF(p)EF) or heart failure with decreased ejection fraction (HF(r)EF), the prevalence of anemia is higher in patients under the age of 85, surpassing 20% [9-11]. Studies have shown that compared to non-anemic patients with HF, anemic patients are older and more likely to be female with diabetes, chronic kidney disease (CKD), severe HF with worsened functional status, decreased exercise capacity, worsened health-related quality of life (QOL), edema, lower blood pressure, the greater requirement of diuretics and higher neurohumoral and pro-inflammatory cytokine activation [12-14]. However, many studies showed that anemic patients have a better left ventricular ejection fraction (LVEF). Hemoglobin is inversely related to LVEF [15,16], an increase in hemoglobin over time is associated with a decrease in LVEF [17]. This was contradictory to our research basis.

Hence, studies have also concluded that the measurement of hemoglobin serves as an accurate indicator of true anemia in the majority of anemia patients with HF [18]. Another similar study examined 148 patients with stable heart failure (HF) and found that a specific cause of anemia was identified in only 43% of the participants. Only 5% of patients had ID, whereas the rest (57%) had chronic disease anemia due to pro-inflammatory cytokine activation, insufficient erythropoietin synthesis, or poor iron utilization. Therefore, chronic disease anemia and an active pro-inflammatory state may be the most common underlying causes of anemia in HF [19]. Recently many studies have linked an increase in inflammatory cytokines like interleukin-1 and (interleukin) IL-6 to an increased risk of coronary heart disease in humans and a worsening of cardiac remodeling in mice [20,21], which is caused by mutation or deficiency of genes that regulate hematopoiesis.

So far, according to many studies, we have a limited number of studies that have shown the use of ID as a standalone prognostic factor in CHF. The comparative impact of ID alone on prognostic outcomes in patients with CHF, stratified by the presence or absence of anemia, has not yet been investigated [22]. So, the goal of our study was to find out how often ID happens, what causes it, and how important it is as a predictor in people with systolic CHF who get care as outpatients, even if they have anemia.

## Materials and methods

Our study adopted a hospital-based, prospective, observational and descriptive (cross-sectional) approach, focusing on patients diagnosed with both chronic heart failure (CHF) and iron deficiency anemia (IDA). The study encompassed a total of 66 patients as participants, and the research spanned a duration of 18 months, from December 1, 2017, to May 31, 2019. The study was carried out at Krishna Hospital and Medical Research Centre, involving patients from both general inpatient wards and the Intensive Care Unit (ICU) specializing in Medicine and Cardiology.

Patients meeting the inclusion criteria were those with ambulatory CHF, classified as New York Heart Association (NYHA) class I, II, or III, with hemoglobin levels ranging from 7 g/dl to 11 g/dl, and patients with systolic HF. Patients aged under 18 years were also included. Conversely, patients with conditions other than iron deficiency anemia, those having rheumatic valvular heart disease or congenital heart disease, patients with impaired liver and renal function, and individuals exhibiting hypersensitivity reactions to intravenous iron were excluded from the study. The sample size can be calculated as sample size=n=4𝑝𝑞/𝑒2, where p=79%=0.79, q=1-p=0.21, considering ‗e‘ absolute errors of 10% n=4×0.79×0.21/0.1×0.1 n=0.66, n=66.

A total of 66 subjects diagnosed with HF seeking treatment in the Department of Medicine Inpatient Department (IPD) and Outpatient Department (OPD) were included and investigated for the presence of IDA. The data was collected during an outpatient or inpatient department visit, recorded in a predesigned and pretested proforma and analyzed. After receiving informed and written consent, all subjects underwent history-taking, examination and routine laboratory investigations. On admission, the following data was collected from each subject: name, age, gender and comorbidities. The examination of subjects also included a general examination and a systemic examination, along with the recording of vitals. Hematological parameters include hemoglobin (Haemometer, Top Tech Bio Medicals Mumbai), serum iron (Roche Cobas c501, USA), total iron binding capacity (TIBC, Beckman Coulter AU480, India), transferrin saturation percentage (TSAT% = (serum iron/TIBC) × 100), left ventricular ejection fraction (LVEF, 2D echocardiography, Nivan Healthcare Solutions, India) and ferritin (Abbott Architect Ferritin Assay, Delhi). Other blood investigations include liver function tests and renal function tests. Other investigations include two-dimensional echocardiography on admission and follow-up and a 12-lead ECG.

Statistical analysis

The obtained data were coded, analyzed and tabulated (Statistical Package for Social Science (SPSS version 16) trial version). Basic descriptive statistical analysis of the quantitative variables was performed in the form of frequencies, means, percentages, standard deviations and the chi-square test. The variables are shown as the number of cases with a percentage mean and standard deviation (±SD). The p-values <0.05 were considered as statistically significant.

Ethical consideration

Before conducting the study, ethical permission was obtained from the ethical committee of Krishna Vishwa Vidyapeeth. The study was assigned an institutional review board number IEC/KVV/2017/22.

## Results

Demographic characteristics of study participants

A total of 66 patients were included in the present prospective observational study. Out of 66 patients, 33 (50%) were males and 33 (50%) were females. A total of four (6.06%) patients were in the age group of less than 30 years; of them, one (3.03%) was male and three (9.09%) were female. A total of 21 (31.82%) were in the age group between 31 and 60 years; of them, 10 (30.30%) patients were males and 11 (33.33%) were females. A total of 41 (62.12%) patients were in the age group of more than 60 years; of them, 22 (66.67%) were males and 19 (57.58%) were females. The youngest patient is 23 years old, and the oldest patient is 87 years old, with an average age of 63.36 years and a standard deviation of 13 years. About 62.12% of patients have an age greater than 60 years (p<0.056), DF=2 and X2=1.267 (Table 1).

**Table 1:** Demographic characteristics of study participants

Age in years	Female		Male		Total	%
	n	%	n	%		
≤30	03	9.09%	01	3.03%	4	6.06%
31-60	11	33.33%	10	30.30%	21	31.82%
>60	19	57.58%	22	66.67%	41	62.12%
Total	33	100%	33	100%	66	100%

Demographic profile and distribution of age with risk factors

The study consisted of 66 participants, with four individuals below the age of 30. Among the participants, one male patient (1%) had a medical history of ischemic heart disease (IHD), while one male and one female patient had a history of tobacco chewing. Additionally, one female patient (1%) had a medical history of hypothyroidism. The remaining participants did not have any significant past medical history. In the age group of 30-60 years, a history of hypertension was present in two (3%) males and three (4.1%) females. History of ischemic heart disease was present in four (6.4%) males and one (1%) female patient. History of type 2 diabetes was present in one (1%) male and three (4.1%) females, tobacco and smoking history was present in five (7.12%) males and eight (12.2%) females and one patient had no past history. In the age group more than 60 years, a history of hypertension was present in which four (6.4%) are males and nine (13.4%) are females; a history of IHD was present in eight (12.2%) males and six (9.3%) females. History of type 2 diabetes mellitus was present in two (3%) males and four (6.4%) females, chronic obstructive pulmonary disease (COPD) was present in two (3%) males and one (1%) female patient, history of tobacco chewing and smoking was present in eight (12.2%) males and four (6.4%) females and five patient had no past history.

**Table 2:** Risk factor

Risk factors	< 30 years		30-60 years		>60 years	
	Male	Female	Male	Female	Male	Female
Ischemic heart disease (IHD)	0	0	2 (3%)	3 (4.1%)	4 (6.4%)	9 (13.4%)
Hypertension	1 (1%)	0	4 (6.4%)	1 (1%)	8 (12.2%)	6 (9.3%)
Type 2 DM (Diabetes mellitus)	0	0	1 (1%)	3 (4.1%)	2 (3%)	4 (6.4%)
COPD (chronic obstructive pulmonary disease)	0	0	0	0	2 (3%)	1 (1%)
Tobacco	1 (1%)	1 (1%)	5 (7.12%)	8 (12.2%)	8 (12.2%)	4 (6.4%)
Other	0	1 (1%)	0	0	0	0
No past history	0	1 (1%)	1 (1%)	0	3 (4.1%)	2 (3%)

Study parameters in patients at the time of admission

Various parameters of heart disease patients were studied at the time of admission. Observed hemoglobin was 8.09 ± 2.17 mg/dl, iron was 34.27 ± 15.03 μg/dl, total iron binding capacity (TIBC) was 294.59 ± 82.44 μg/dl, transferrin saturation percentage (TSAT%) was 11.83 ± 6.50, ferritin was 17.94 ± 5.41 ng/dl and LVEF was 42.39% ± 11.78% (Table 3).

**Table 3:** Parameter (at the time of admission) TSAT%: transferrin saturation percentage, SD: Standard deviation.

Parameters	Mean	SD (±)
Hb (hemoglobin)	8.09	2.17
Iron	34.27	15.03
TIBC (total iron binding capacity)	294.59	82.44
TSAT%	11.83	6.50
Ferritin	17.94	5.41
LVEF (left ventricular ejection fraction)	42.39%	11.78%

Changes in study parameters before and after treatment

Two parameters were studied before and after treatment, hemoglobin and LVEF. For comparison of improvement in LVEF and hemoglobin, before and after treatment paired t-test was used. Observed hemoglobin before and after treatment was 8.09 ± 2.17 and 10.29 ± 1.05, respectively, observed improvement in hemoglobin was significant (8.09 versus 10.29, p<0.0001) and the percentage change was 27.19%. Observed LVEF before and after treatment was 42.39 ± 11.78% and 47.21 ± 9.99%, respectively, observed improvement in LVEF was significant (42.39% versus 47.21%, p=0.012) and percentage change was 11.37% (Table 4).

**Table 4:** Change in parameter after treatment LVEF: left ventricular ejection fraction.

Parameters	Before		After		p-value	Percentage change in values after treatment
	Mean	SD	Mean	SD		
Hemoglobin	8.09	2.17	10.29	1.05	<0.0001	27.19%
LVEF	42.39%	11.78%	47.21%	9.99%	0.012	11.37%

NYHA class before and after treatment

In the present study, after treatment NYHA classification showed significant improvement (p-value <0.001). Before the intervention, 57.58% of patients had NYHA class II and 42.4% of patients had NYHA class III. After treatment, 33.33% of patients showed NYHA class II and 19.70% of patients showed NYHA class III. After Iron therapy treatment, out of 29 cases of NYHA class III, nine (31.03%) cases converted to NYHA class I, seven (24.14%) cases converted to NYHA class II and 13 (44.83%) cases belong to the same NYHA class. Out of 37 cases of NYHA class II, 22 (59.45%) cases converted to NYHA class I and 15 (40.54%) cases belong to the same NYHA class (Table 5).

**Table 5:** NYHA class (before and after treatment) NYHA: New York Heart Association.

NYHA class (before treatment)	NYHA class (after treatment)			
	No symptoms	I	II	III
I 15 (22.72%)	14 (21.21%)	1 (1%)	0 (0%)	0 (0%)
II 28 (42.42%)	15 (22.72%)	13 (19.69%)	0 (0%)	0 (0%)
III 23 (34.84%)	2 (3.03%)	9 (13.63%)	12 (18.18%)	0 (0%)
Total 66 (100%)	31 (46.96%)	23 (34.48%)	12 (18.18%)	0 (0%)

## Discussion

Our research team conducted a study that yielded favorable results in improving the New York Heart Association (NYHA) class when intravenous iron was administered for a duration of 24 weeks. The observed benefit was evident at the four-week mark and persisted for the duration of the whole study. The observed enhancements in hemoglobin levels and left ventricular ejection percent were found to be uniform across all predetermined groups, thereby validating these results. The various studies that can be compared are illustrated in Table 6 [23-30].

**Table 6:** Comparison of various studies with our study HTN: hypertension, LVEF: left ventricular ejection fraction, IHD: ischemic heart disease, Hb: hemoglobin.

Study name	Males	Mean age (years)	Comorbidity	Hb before treatment	Hb after treatment	LVEF Before treatment	LVEF after treatment	p-value for change in Hb	p-value for change in LVEF
Okonko et al. (2008) [23]	60%	64	IHD (79%)	12.2	12.6	30%	32%	<0.87	0.66
Bolger et al. (2006) [24]	75%	68.3	IHD (65%)	11.2	12.6	26%	33%	<0.002	=0.001
Silverberg et al. (2000) [25]	79%	70.1	HTN (71%)	10.16	12.10	27.7%	35.4%	<0.05	<0.002
Ponikowski et al. (2015) [26]	68%	60.2	HTN (60%)	12.37	14.20	37.1%	39.2%	<0.001	<0.005
Rangel et al. (2014) [27]	69%	63.2	Dyslipidemia (76%)	13.7	14.3	26.7%	35%	<0.001	<0.001
Toblli et al. (2007) [28]	64%	65.2	IHD (62%)	12.5	14.9	30.1%	32.4%	<0.004	<0.001
van Veldhuisen et al. (2011)[29]	75%	64	IHD (57%)	11.4	12.8	36.9%	40.1%	<0.001	<0.004
Anker et al. (2009) [30]	52.3%	67.8	IHD (79.9%)	11.9	14.6	31.9%	36.2%	<0.002	<0.04
Present study	50%	63.36	IHD (30.30%)	8.09	10.29	42.39%	47.21%	<0.0001	=0.002

In this regard, among all study populations, two parameters were studied before and after treatment: hemoglobin and LVEF. Observed hemoglobin was 8.09 ± 2.17 mg/dl, iron was 34.27 ± 15.03 μg/dl, TIBC was 294.59 ± 82.44 g/dl, TSAT% was 11.83 ± 6.50, ferritin was 17.94 ± 5.41 ng/dl and LVEF was 42.39% ± 11.78%. NYHA class I was found in 15 (22.72%), class II in 28 (42.42%) and class III in 23 (34.84%). In our study before treatment, the most commonly observed symptom was easy fatigability (66%), followed by breathlessness and exertion (24 cases, 36.36%), and preceding that, 14 (21.21%) cases had palpitation and 14 (21.21%) cases had pedal edema. It has been well demonstrated that anemia is a powerful factor for predicting adverse outcomes in these patients, so anemia is concomitant with other risk factors. In our study, the most commonly observed past history was IHD (20 cases, 30.30%), followed by hypertension (19 cases, 28.79%), type 2 diabetes mellitus (10 cases, 15.15%), chronic obstructive pulmonary disease (three cases, 4.54%) and others such as tobacco chewing or smoking (27 cases, 39.92%), which may have synergistic effects to deteriorate the outcome of disease. Hence, a correct diagnosis can easily be arrived at using parameters such as serum ferritin and transferrin saturation.

The study limitations include a relatively small sample size of 66 subjects, potentially limiting the generalizability of the findings to a broader population. The study was conducted in a single medical department, which might limit the diversity of patient profiles and the generalizability of results to other healthcare settings. The long-term impact of iron therapy and potential relapses in NYHA classification over extended periods were not explored. The study lacks a control group for comparison, making it challenging to determine if the observed improvements in NYHA classification are solely due to iron therapy or could be influenced by other factors. The assessment of NYHA classification might be influenced by the participants' and medical staff's expectations or perceptions, introducing a potential bias in the reported results.

## Conclusions

Overall, our study underscores the importance of addressing iron deficiency anemia in individuals with systolic chronic heart failure. Iron therapy exhibited a significant positive impact on the patient's functional status, as evidenced by the improvement in NYHA classification. These findings provide valuable insights into the potential benefits of incorporating iron therapy into the management of CHF patients receiving outpatient care. Further research and clinical trials are warranted to confirm and expand upon these encouraging results.
